# Landscape simplification increases vineyard pest outbreaks and insecticide use

**DOI:** 10.1111/ele.13622

**Published:** 2020-10-13

**Authors:** Daniel Paredes, Jay A. Rosenheim, Rebecca Chaplin‐Kramer, Silvia Winter, Daniel S. Karp

**Affiliations:** ^1^ Department of Wildlife Fish and Conservation Biology University of California Davis CA USA; ^2^ Department of Entomology and Nematology University of California Davis CA USA; ^3^ Natural Capital Project Stanford University Stanford CA USA; ^4^ Institute of Plant Protection University of Natural Resources and Life Sciences Vienna Austria

**Keywords:** Biological control, ecoinformatics, ecosystem services, integrated pest management, *Lobesia botrana*, Spain

## Abstract

Diversifying agricultural landscapes may mitigate biodiversity declines and improve pest management. Yet landscapes are rarely managed to suppress pests, in part because researchers seldom measure key variables related to pest outbreaks and insecticides that drive management decisions. We used a 13‐year government database to analyse landscape effects on European grapevine moth (*Lobesia botrana*) outbreaks and insecticides across *c*. 400 Spanish vineyards. At harvest, we found pest outbreaks increased four‐fold in simplified, vineyard‐dominated landscapes compared to complex landscapes in which vineyards are surrounded by semi‐natural habitats. Similarly, insecticide applications doubled in vineyard‐dominated landscapes but declined in vineyards surrounded by shrubland. Importantly, pest population stochasticity would have masked these large effects if numbers of study sites and years were reduced to typical levels in landscape pest‐control studies. Our results suggest increasing landscape complexity may mitigate pest populations and insecticide applications. Habitat conservation represents an economically and environmentally sound approach for achieving sustainable grape production.

## Graphical abstract

Diversifying agricultural landscapes may mitigate biodiversity declines and improve pest management. We used a 13‐year government database to analyse landscape effects on European grapevine moth (Lobesia botrana) outbreaks and insecticides across *c*. 400 Spanish vineyards. Our results suggest increasing landscape complexity may mitigate pest outbreaks and insecticide applications.

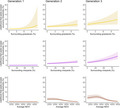

## INTRODUCTION

Agricultural expansion and intensification have increased food production (Foley *et al*., 2005), but also contributed to the on‐going biodiversity crisis (Dirzo *et al*., 2014). Because cultivating crops in monoculture creates the perfect conditions for specialist pest outbreaks (Andow, 1983), farmers have consistently turned to insecticides to maintain high yields under constant pest pressure.

However, new insecticides must be constantly developed as pests evolve resistance (Gould *et al*., 2018). Some of these products are known to compromise human health (Bouchard *et al*., 2011) and cause declines in biodiversity (Köhler and Triebskorn, 2013) and ecosystem services (e.g. pollination; Whitehorn *et al*., 2012). Moreover, broad‐spectrum insecticides can reduce populations of predators and parasitoids, releasing pests from top‐down control and allowing populations to resurge (Pimentel *et al*., 1992).

Integrated pest management (IPM) was developed to provide growers with a broader toolkit for preventing pest outbreaks while simultaneously reducing their reliance on insecticides. The aim of IPM is not to eradicate pests, but rather to maintain populations below crop injury levels (i.e. economic thresholds) at which crop damage would be substantial enough to justify insecticide applications (Stern *et al*., 1959). Although IPM often focuses on local (farm‐level) interventions, ecologists, agronomists and farmers are increasingly recognising the critical role that surrounding landscapes can play in determining pest damage (Thies and Tscharntke, 1999; Bianchi *et al*., 2006).

At least three mechanisms that can operate simultaneously may underlie landscape effects on pests. First, the resource concentration hypothesis posits that simple landscapes (i.e. expansive crop monocultures) allow specialist pest populations to build and disperse, whereas complex landscapes (i.e. mosaics of semi‐natural habitat and cropland) mitigate population growth and spread (Root, 1973; O’Rourke and Petersen, 2017). Second, parasitoids and predators of crop pests often depend on multiple crops and/or natural habitats for alternate food resources or as overwintering habitats (Landis *et al*., 2000). Thus, complex landscapes may augment the abundance and/or diversity of natural enemies, facilitating better pest control (Bianchi *et al*., 2006; Chaplin‐Kramer *et al*., 2011; Dainese *et al*., 2019). Third, pests may themselves depend on non‐crop habitats to overwinter or feed on alternative host plants (Tscharntke *et al*., 2016).

Perhaps because these mechanisms may operate to varying degrees for different pests in different systems, recent work suggests that pest responses to landscape composition are quite variable across studies (Karp *et al*., 2018). In many studies, pests are best controlled in complex landscapes with patches of natural habitat (e.g. Thies and Tscharntke, 1999). In others, pests thrive in complex landscapes (e.g. Midega *et al*., 2014). Similarly, some studies report fewer insecticides being applied in landscapes with more natural vegetation and crop diversity (Meehan *et al*., 2011; Larsen and Noack, 2017), while others report significant variability in landscape effects over space and time (Larsen, 2013, but see also Meehan and Gratton, 2015).

Importantly, many pest‐control studies may not be conducted at large enough spatio‐temporal scales to tease apart landscape effects from the inherent variability of pest population dynamics. To feasibly monitor pests and natural enemies at a landscape scale, most studies are conducted over 10–30 sites for 1–2 years, relying on only a few instantaneous population measurements each year (Karp *et al*., 2018). Yet, pest population dynamics are often highly stochastic (Murdoch *et al*., 1985), with abundances fluctuating both within and between years. As such, failing to monitor pests throughout the growing season may obscure population dynamics and mask landscape effects (Chaplin‐Kramer *et al*., 2013). Similarly, failing to sample across broad spatial scales, for multiple years, may miss the rare but severe outbreaks that farmers care about (Chaplin‐Kramer *et al*., 2019). In addition, most landscape studies focus on broad land‐cover classes rather than remotely sensed measures that could more directly encapsulate pests’ niches (e.g. landscape productivity or vegetation structure; Ramirez‐Reyes *et al*., 2019).

Finally, agroecologists rarely measure the key pest‐control variables that drive land management decisions (Chaplin‐Kramer *et al*., 2019). Most studies focus on natural enemies, and the fewer studies that focus on pests usually quantify relative pest abundances between sites (Bianchi *et al*., 2006; Gurr *et al*., 2016). For farmers, relative abundances are less relevant than knowing whether pests are likely to exceed established economic injury levels and damage crops (Gurr *et al*., 2016). For governments and non‐governmental organisations concerned about environmental and/or human health, it is critical to know whether landscape effects are strong enough such that effective landscape management could reduce insecticide application rates (European Parliament, 2009; Meisner *et al*., 2017). Furthermore, even if pests are suppressed below insecticide thresholds, it is unclear how often farmers actually reduce insecticide use versus continuing to apply insecticides prophylactically (Liu and Huang, 2013; Sogawa, 2015). Without this information, it is unsurprising that landscapes are very rarely managed with pest control in mind (Chaplin‐Kramer *et al*., 2019).

Here, we explore the effects of surrounding landscape composition on European Grapevine Moth (*Lobesia botrana*, Lepidoptera: Tortricidae) infestations using an ‘ecoinformatic’ approach (i.e. analysing a large, pre‐existing database rather than collecting field data across a more limited number of sites; Rosenheim and Gratton, 2017). Specifically, we acquired and analysed a government‐sponsored database of *c*. 400 vineyards monitored for *c*. 13 years in Southern Spain. In Spain, *L. botrana* completes three generations within each growing season. In the first generation, the pest feeds on flowers. In the second and third generations, adults lay eggs on grapes and the developing larvae consume the berries, leading to substantial damage (Moschos, 2006). Although it is one of the most important and widespread vineyard pests, *L. botrana* is polyphagous (Thiéry and Moreau, 2005). Indeed, there is some evidence that the pest’s fitness peaks when feeding on several native and cultivated species that may exist in the semi‐natural habitats that surround European vineyards (Thiéry and Moreau, 2005). At the same time, surrounding semi‐natural habitat may indirectly depress *L. botrana* populations through increasing natural pest control by birds and predatory arthropods (Rusch *et al*., 2017; Papura *et al*., 2020). *L. botrana* thus constitutes an excellent candidate for an ‘ecoinformatic’ approach to pest modelling: potentially opposing direct and indirect landscape effects on *L. botrana* may precipitate mixed or non‐significant effects in more typical, shorter‐term and less well‐replicated pest‐control studies.

**Figure 1:**
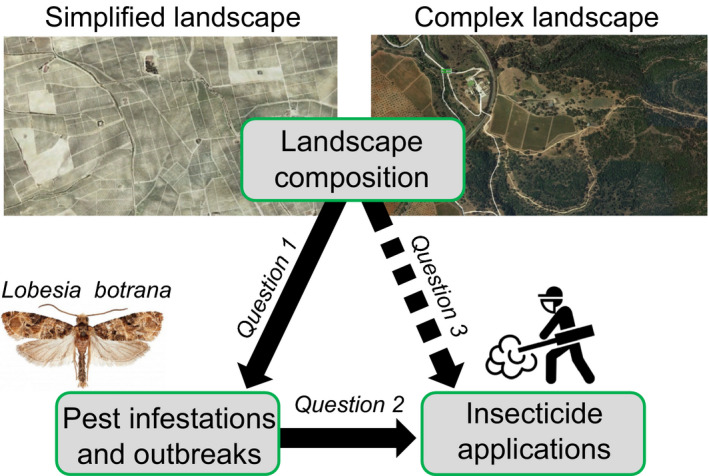
Conceptual diagram. Figure depicts our three guiding questions, relating (1) landscape composition with pest infestations and outbreaks, (2) pest outbreaks with farmer decisions about how often to apply insecticides and (3) landscape composition with insecticide application rates. Direct effects are solid arrows; indirect effects are dotted arrows.

We organised our study around three guiding questions (Fig. 1). First, to what extent does landscape simplification exacerbate pest infestations? Specifically, are landscape effects strong enough to affect the probability that pests exceed established economic injury thresholds? Second, do farmers follow IPM guidelines, applying insecticides more often when pests exceed economic thresholds? Third, how do insecticide application rates vary between farms located across different landscape contexts? In all cases, we were also interested in quantifying spatiotemporal stochasticity and determining whether landscape effects would still be apparent in more traditional studies with fewer sites monitored for fewer years.

## MATERIAL AND METHODS

### RAIF database

**Figure 2:**
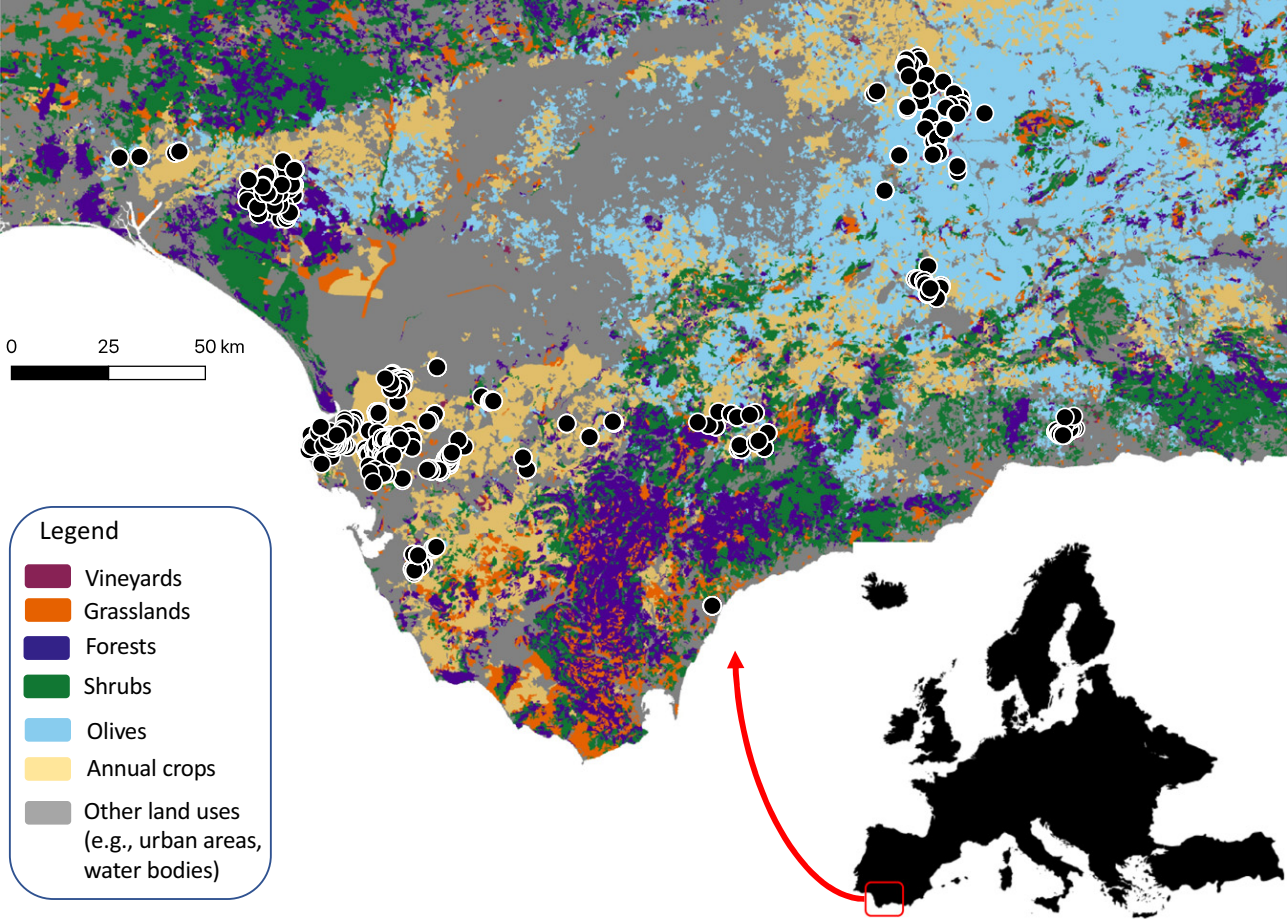
Study site map. Map depicts vineyard study sites (black dots) in the Andalusia region of Southern Spain. Inset shows study region location within Europe.

We obtained a large database of pest densities over 13 years (2006–2018) in Andalusia, Spain (Fig. 2). This database was provided by the Andalusian Government through the RAIF (Red de Alerta e Información Fitosanitaria) network. The RAIF database is unrivalled in its duration, detail and level of replication, offering much more data than typical pest‐control studies. The RAIF network monitors pest populations on private vineyards throughout Andalusia, providing guidelines to farmers on when and how to treat pest outbreaks. In all subsequent analyses, we consider a ‘vineyard‐year’ to be our unit of replication (i.e. each vineyard surveyed in each year constitutes a sampling unit).

Since 2006, RAIF technicians have visited most focal vineyards on a weekly basis throughout the growing season to collect data on *L. botrana* infestations and vineyard management, including agrochemical applications. *L. botrana* infestations were measured by quantifying the proportion of grape inflorescences or bunches (of 100) with eggs present. Technicians also consult with farmers to acquire the date of application, dose, target pest and product name of each insecticide, herbicide or fungicide application. All farmers purportedly use IPM criteria, wherein insecticides are only applied when pest populations exceed relevant economic injury thresholds (in this case, 8% of grape bunches with eggs; BOJA, 2005). Finally, technicians record soil tillage frequencies, irrigation management (i.e. whether vineyards were irrigated or not) and grape cultivars.

We supplemented the RAIF database with topographic information (elevation, slope and aspect) and the regional climate associated with each farm. Topographic information was extracted from a Digital Elevation Model (Instituto Geográfico Nacional, 2019). For climate data, we leveraged 79 weather stations located throughout the study region. We first calculated average monthly temperature and precipitation values at the weather station closest to each farm. We then conducted two principal components analyses (for temperature and for precipitation) and extracted the first two axes for each analysis. The two axes explained 54% and 58% of the temperature and precipitation variation respectively.

### Landscape context

To explore landscape effects on pest outbreaks and management, we extracted information on the land cover surrounding each farm from the CORINE Land Cover Inventory. Although our focus was on landscape composition, we recognise that habitat configuration may also influence pest population dynamics and deserves further study (Martin *et al*., 2019). Here, however, we used CORINE land‐cover maps to quantify the amount of forest, shrublands (i.e. sclerophyllous vegetation and transitional woodland shrubs), grasslands (i.e. natural grasslands, pastures, moors and heathlands), vineyards, olive groves and annual crops (viz., cereals) surrounding each site.

Despite being one of the most accurate and highly resolved land‐cover products, the CORINE data layer’s spatial resolution of 100 m still means many small vineyards may be misclassified. Indeed, 23% of our vineyard sites were classified neither as vineyards nor on the border of vineyards in the CORINE data layer. After comparing these sites with Google Earth imagery, we discovered 9% of our sites were located further than 100 m from any visible vineyard, indicating that the farms’ coordinates were incorrect. These sites were excluded from analyses. The remaining 14% of sites were clearly located in vineyards based on Google Earth imagery; however, these vineyards were significantly smaller than vineyards that were identified correctly in CORINE (average of 4.98 ha vs. 12.20 ha; *t*‐test *P*‐value <0.001). As such, our calculations of surrounding vineyard cover may be slight underestimates.

In addition to land cover, we also calculated the landscape productivity surrounding each site. Specifically, we used Landsat 7 (combined with Landsat 8 for 2018)‐derived annual composites of the normalised difference vegetation index (NDVI) for each year (available on Google Earth Engine). Annual mean NDVI was calculated for each year beginning on September 15, corresponding to the end of the third generation of the *L. botrana*. Ten vineyards were located close to the ocean (>10% water within 2 km) and thus excluded from subsequent analyses.

To calculate proportional land cover and productivity values around each site, we calculated indices that disproportionally weight landscape elements located closer to focal farms. To do so, we first calculated the proportion of each land cover and average NDVI in a series of concentric rings (100 m widths) up to 2 km from each farm location. Then, following Karp *et al*. (2016), we calculated a weighted average of all the rings surrounding each site, using Gaussian decay functions with three different decay rates (250, 750 and 1250) to vary the relative influence of areas closer versus farther away from the farm. As CORINE land‐cover products are not available for each year, we linearly interpolated values calculated from the 2006, 2012 and 2018 maps to derive landscape composition estimates for each year of the study. For example, for data collected in 2008, we averaged values obtained from the 2006 to 2012 maps, giving twice the weight to the 2006 land‐cover map. In later analyses, we compared Akaike Information Criteria (AIC) between models with different decay rates, finding 1250 to be generally most predictive (Table S1 and S2). We, thus, present results using the 1250 decay rate, but also include analyses using other decay rates as supplementary tables and figures.

### Modelling

We used Generalised Additive Mixed Models (GAMM) to assess landscape effects on pest infestations, outbreaks and insecticide applications. We chose GAMMs as they are versatile, allowing for non‐linear relationships between response variables and predictors (Zuur *et al*., 2007). Their ability to accommodate random effects also makes it possible to account for spatiotemporal dependence and observer effects (thereby, avoiding pseudoreplication). We first used a GAMM to predict variation in pest infestation rates over the growing season. The resulting model confirmed that *L. botrana* completes three generations each growing season, with infestation rates sequentially increasing from the first to the third generations (Fig. S1). We used our model to define each generation, and then conducted separate analyses of pest infestations and outbreaks for each generation. Sample sizes varied by generation (generation 1 – March–May, 342 vineyards, 804 vineyard‐years; generation 2 – May–July, 412 vineyards, 1123 vineyard‐years; and generation 3 – July–September; 387 vineyards, 1052 vineyard‐years).

We then calculated three distinct response variables. First, we modelled the average percentage of grape inflorescences (generation 1) or bunches (generations 2 and 3) infested with *L. botrana* across all farm visits within each generation in each year, using a negative binomial distribution. Second, we modelled the likelihood that each farm would experience an outbreak by first determining whether the pest exceeded the economic injury threshold (>8% bunches infested) for each farm visit. We then used binomial distributions to model the number of farm visits for which the pest exceeded versus did not exceed the threshold within each pest generation. Finally, we used Poisson distributions to model the total number of insecticide applications specifically targeted towards *L. botrana* across all three generations within the growing season. Model assumptions were not violated when using these distributions (we avoided heteroskedasticity and overdispersion).

We tested for collinearity before formulating the models’ fixed‐effect structures. High correlations (*r* > 0.5) occurred between slope and altitude. We therefore excluded slope, as it appeared less predictive than altitude in exploratory analyses. As agricultural land uses were also collinear, we decided to focus on surrounding vineyard cover in the main text. Olive orchard cover never yielded significant effects (Table S3). Although annual crop cover effects were occasionally significant, vineyards and annual crops were negatively correlated (Fig. S2 and models including vineyard cover were always better supported (i.e. lower AIC and more deviance explained; Table S3). Thus, our final set of fixed effects included landscape variables (mean NDVI as well as the proportion of surrounding forest, shrubland, grassland and vineyard cover) as well as key covariates related to topography (altitude and aspect) and regional climate (first two PC axes for temperature and precipitation). As a final check that collinear landscape predictors did not cause spurious trends, we also fitted models that only included one landscape predictor at a time.

Because insecticide analyses suffer from issues surrounding bidirectional causality (i.e. insecticides may reduce pest infestations, or high pest infestations may trigger the application of insecticides), we conducted two sets of analyses in which we included versus excluded an additional set of farm management variables (i.e. number of insecticide, herbicide and fungicide applications; number of tillage events and the presence of irrigation). We present results from models without farm management; however, results were very similar when management variables were included. Finally, after conducting exploratory analyses in which spatial splines for each fixed effect were left unconstrained (i.e. unlimited numbers of knots), we decided to set the maximum number of knots to three for each predictor to avoid overfitting (Taylan *et al*., 2007).

Many, but not all, farms were surveyed on a weekly basis. As such, numbers of farm visits per generation varied across vineyards and study years. For each generation in each year, we excluded all farms visited fewer than four times. For those models that use data across the entire season, we excluded farms visited fewer than ten times. In most models, we also included the number of farm visits as a weighting factor to account for sample size variation among farms. The one exception was for models assessing the likelihood that pests exceeded an economic threshold, as this information was encapsulated in the response variable (i.e. a binomial model assessing the number of visits exceeding versus not exceeding economic thresholds).

Models shared similar random effect structures. Specifically, we included random effects of survey year (*n* = 13 years), observer identity (*n* = 59 technicians), cultivar types (*n* = 14 cultivars) and farm identity (*n* = 475 farms). We also included a random effect of ‘region’ to account for spatial non‐independence among sites (*n* = 17 regions; average of 63 farms/region). This variable was included in the RAIF database and delineates regions with similar crop and pest characteristics. After including region and farm identity effects, no models displayed evidence of spatial autocorrelation (*P* > 0.05 for all Moran’s I tests).

We used two approaches to assess whether exceeding economic thresholds caused farmers to increase insecticide application rates. For the first approach, we modelled the number of insecticide sprays across the entire growing season based on whether or not pests ever exceeded an economic threshold. For the second approach, we modelled insecticide application frequencies as a function of the fraction of farm visits for which the pest exceeded the economic threshold.

We quantified spatiotemporal stochasticity and model adequacy by calculating the deviance explained by each model, both with and without the random effects (to determine the degree to which fixed effects explained variation in response variables). We then used simulations to determine whether smaller sample‐sized studies would have been able to detect landscape effects. Specifically, for 500 iterations, we randomly selected two sequential years and 25 farms, sampling independently across five levels of surrounding vineyard cover to ensure that farms still spanned landscape gradients. Then, for each iteration, we modelled effects of surrounding vineyard cover on pest outbreaks and insecticide application rates, simplifying our prior model structure to avoid overfitting (i.e. excluding other landscape variables, reducing the number of knots for climate/topographic variables to 2, including study year as a fixed effect and omitting all other random effects). All analyses were conducted in R (R Development Core Team, 2018); GAMMs were implemented with the “mgcv” package (Wood, 2011).

## RESULTS

### Landscape effects on pest outbreaks

**Figure 3:**
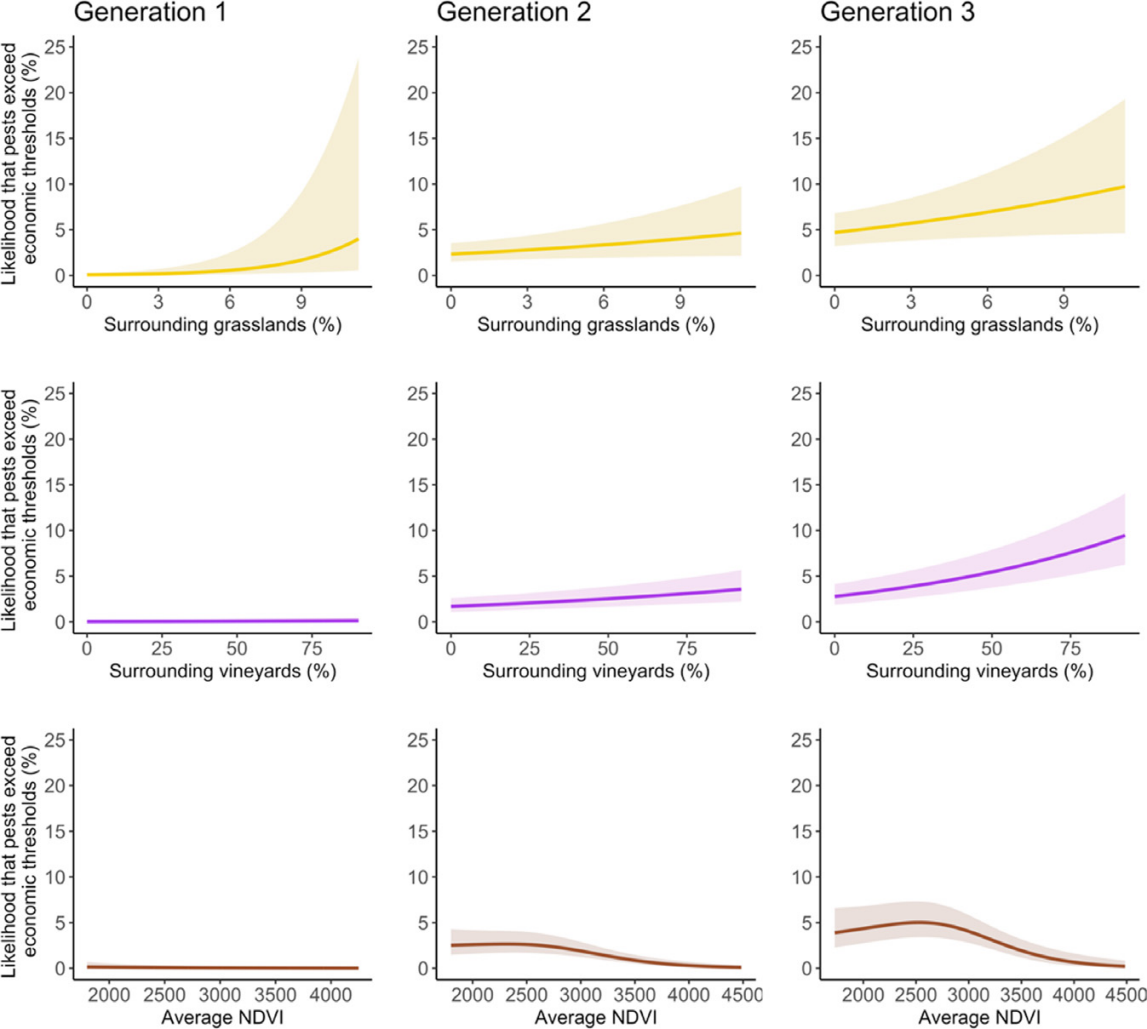
Landscape effects on the likelihood of pests exceeding economic thresholds for each generation of *Lobesia botrana*. Thresholds were more likely to be exceeded in landscapes with more surrounding grasslands in the first but not the second or third generations (top panels; yellow lines). In contrast, farms in landscapes with more vineyard cover were predicted to experience more outbreaks in the second and third generations, but not the first (middle panels; purple lines). Finally, in the second and third generations, thresholds were most likely to be exceeded in low to moderately productive landscapes, measured with the Normalized Difference Vegetation Index (bottom panels; brown lines). Lines represent predictions from GAMMs; shaded regions correspond to 95% confidence regions. X‐axis values are calculated as weighted averages of land‐cover percentages or NDVI values within concentric rings surrounding each study site (using a 1250 Gaussian decay rate; see methods).

Landscape effects on the likelihood that pests exceeded economic thresholds (8% of bunches infested) varied by generation (Fig. 3). Vineyards with more surrounding grassland cover were more likely to experience pest outbreaks above the economic threshold in the first generation (*P* = 0.01; Fig. 3; Table S4), but not in the second (*P* = 0.30) or third (*P* = 0.27) generations. In contrast, farms with more surrounding vineyard cover were more likely to exceed economic thresholds in the second (*P* = 0.02) and third generations (*P* < 0.01), but not the first (*P* = 0.25).

While surrounding forest and shrub cover did not influence the likelihood of surpassing economic thresholds, landscape productivity exhibited non‐linear effects (Fig. 3). At low to moderate levels of mean annual NDVI, the likelihood that pests exceeded economic thresholds was fairly stable at *c*. 2.5% in the second generation and *c*. 5% in the third generation. Higher NDVI values (>2800), however, caused the likelihood of exceeding thresholds to sharply decline. This effect was strongest in the second generation (*P* = 0.04), marginal in the third generation (*P* = 0.09) and absent in the first (*P* = 0.34). Analyses focused on pest infestation levels (rather than probabilities of exceeding economic thresholds) yielded broadly similar results (Table S5; Fig. S3). However, surrounding grassland and vineyard effects on pest infestation levels were significant in every generation. Moreover, infestations decreased with surrounding shrub cover rather than landscape productivity.

### Farmer decisions to apply insecticides

**Figure 4:**
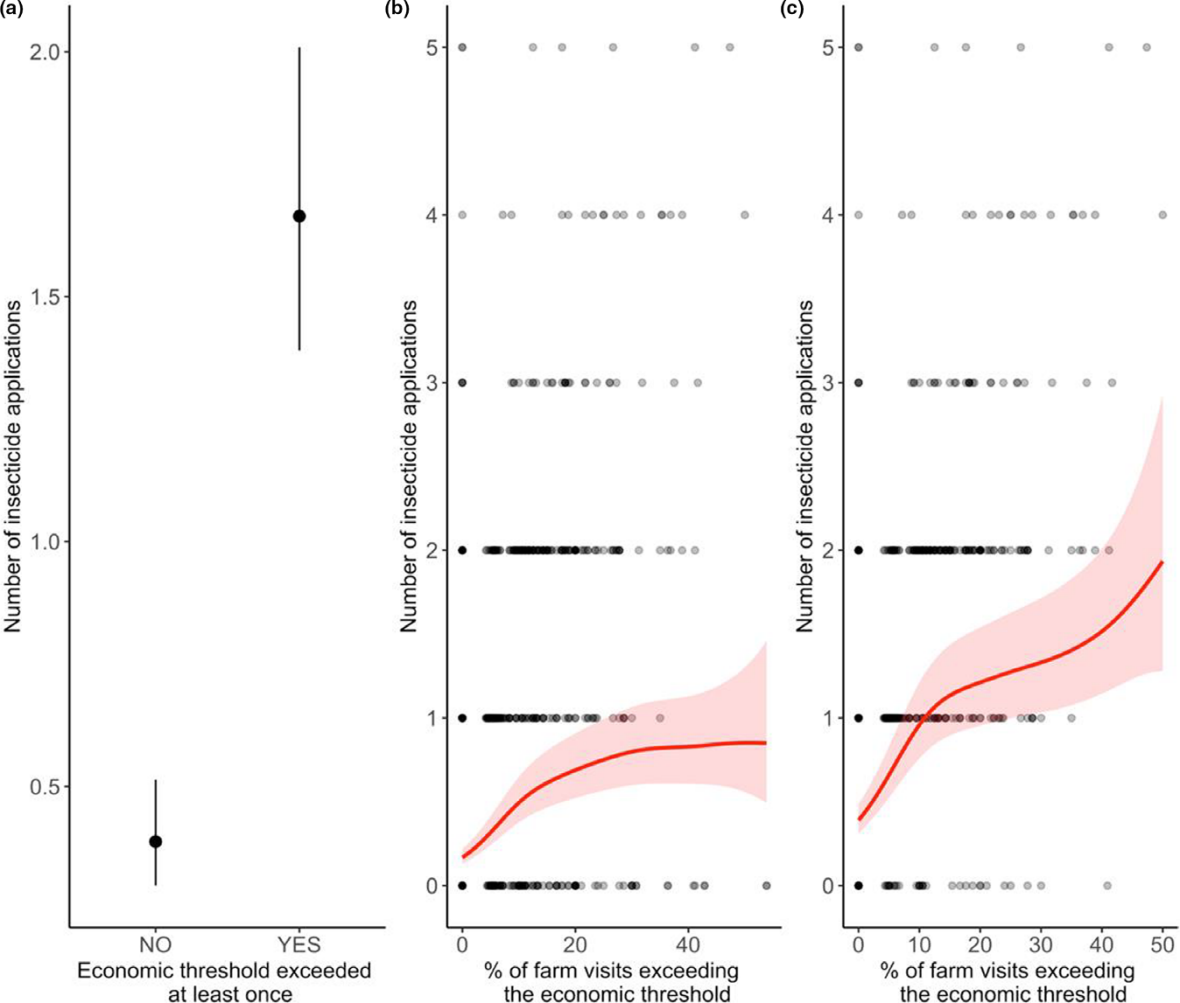
Relationships between economic thresholds and insecticide application rates. Farmers were more likely to apply more insecticides when *Lobesia botrana* infestations exceeded the economic threshold at least once within the growing season (Panel A). However, the fraction of farm visits for which economic thresholds were exceeded exhibited a nonlinear relationship with the number of insecticide sprays (Panel B). When farms that never applied insecticides were excluded (including insecticides targeted to other pests), the trend became almost linear (Panel C). For panel A, points represent average predicted numbers of insecticide sprays from GAMMs; lines are 95% confidence intervals. For panels B & C, lines represent GAMM predictions; shaded regions are 95% confidence regions.

Farmers applied more insecticides if pests exceeded economic thresholds at least one time across the growing season (*P* < 0.01; Fig. 4a; Table S6). Surprisingly, however, the fraction of time spent above the economic threshold exhibited a saturating relationship with spray frequencies (*P* < 0.01; Fig. 4b; Table S7). This nonlinear trend was seemingly driven by some farmers that chose to never apply insecticides, no matter the pest infestation level. Indeed, after excluding farms where no insecticides were applied (including insecticides targeted towards other pests), insecticide application rates increased nearly linearly with the fraction of time spent above the economic threshold (*P* < 0.01; Fig. 4c; Table S8).

### Landscape effects on insecticide applications

**Figure 5:**
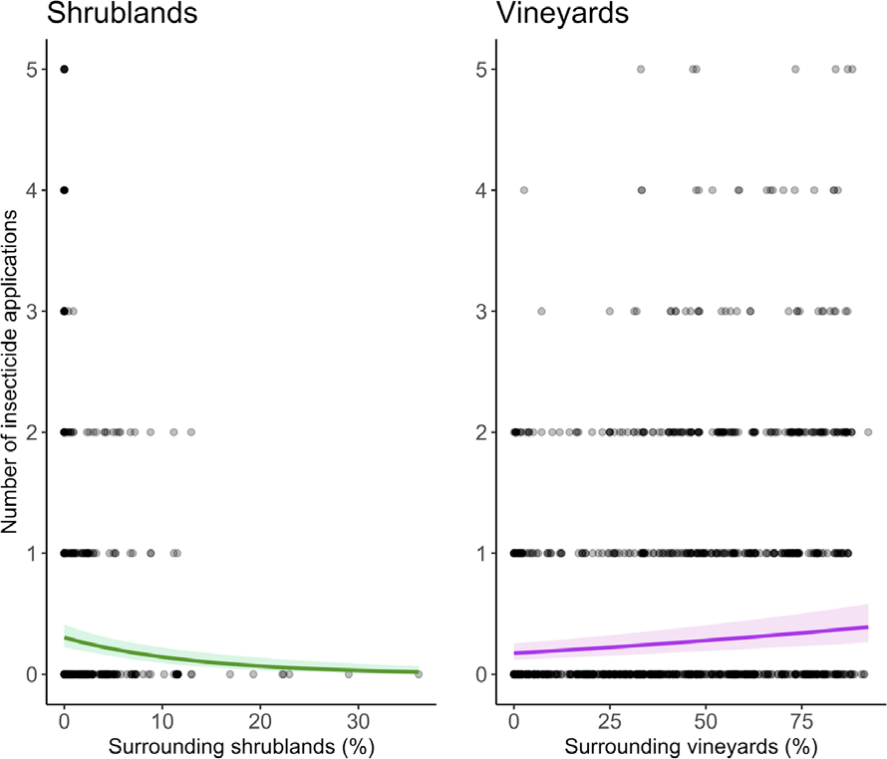
Landscape effects on insecticide application rates. Farmers were more likely to spray insecticides in landscapes with less surrounding shrubland (left panel; green line). In contrast, farmers sprayed more insecticides in landscapes with more surrounding vineyards (right panel; purple line). Lines represent predictions from GAMMs; shaded regions correspond to 95% confidence regions.

Effects of surrounding landscape composition on insecticide application frequencies tended to parallel effects on infestation rates and outbreaks (Fig. 5; Table S9). Farmers sprayed more insecticides when their farms were surrounded by more vineyards (*P* < 0.01) and fewer insecticides when their farms were surrounded by more shrubland cover (*P* = 0.02). Landscape productivity (i.e. NDVI), forest cover and grassland cover) did not affect insecticide applications (Table S9).

### Stochasticity and the importance of long‐term datasets

Random effects of farm location, geographic region and year were often the most important predictors, highlighting the stochasticity of pest populations. Indeed, economic threshold models with random effects explained more than twice the deviance of models without them (with random effects: 75.1, 58.3 and 61.3% of the deviance for generations 1, 2 and 3; without random effects: 29.9, 19.7 and 22.8%). Similar results were observed for models of insecticide application rates (with random effects: 47.1, 54.5 and 52.4% for generations 1, 2 and 3; without random effects: 24.0%, 28.5% and 33.3%).

**Figure 6:**
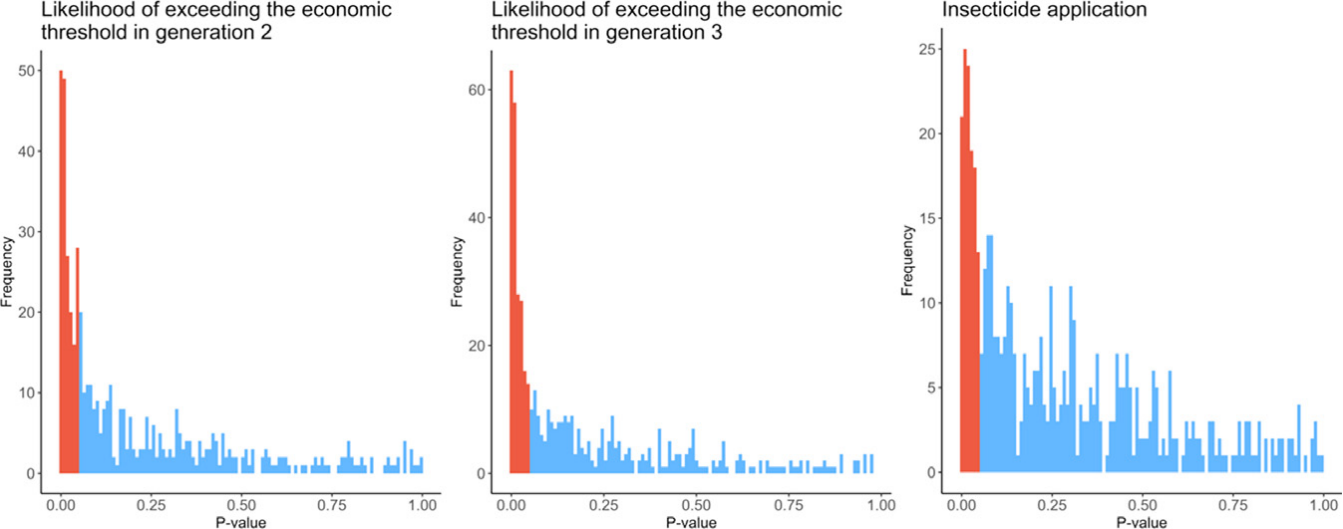
Simulations exploring our ability to detect effects of surrounding vineyard cover on pest outbreaks and insecticide applications in reduced datasets. Histograms display P‐values of surrounding vineyard cover effects on the likelihood of exceeding economic thresholds in generation 2 (left panel) and generation 3 (middle panel) as well as on insecticide application rates (right panel). Surrounding vineyard effects were assessed on reduced datasets, whereby 2 survey years and 25 focal farms were selected randomly 500 times. Simulations in which vineyard effects were significant (*P* < 0.05) are coloured red; blue represents non‐significant vineyard effects.

This high spatiotemporal stochasticity would likely have masked landscape effects in more traditional landscape pest‐control studies. After subsetting the database to 25 farms and 2 study years, *L. botrana* outbreaks (i.e. likelihood of exceeding the economic threshold) were significantly associated with vineyard cover in only 38% and 41% of the simulations (for generations 2 and 3, respectively; Fig. 6). Similarly, vineyard cover was significantly associated with insecticide applications in only 24% of simulations. Removing the fixed effect of year did not influence these trends.

### Covariates and robustness of trends

Although principal components analysis only explained moderate levels of climate variation among sites, temperature and precipitation PC axes still correlated with pest infestations and outbreaks (but not insecticide applications; Fig. S4). Topographic variables (i.e. altitude and aspect) were not important in driving pest outbreaks or insecticide applications.

All results were fairly robust across a variety of model specifications. For example, although some effects changed with respect to the level of significance, effect sizes and signs of landscape effects were broadly consistent across analyses conducted at different Gaussian decay rates (Table S10‐S12). Similarly, results did not appreciably change when farm management variables (insecticide, herbicide, fungicide and tillage frequencies) were included as additional fixed effects (Fig. S5‐S7; Table S13‐S15). One exception was landscape productivity: when calculated at 250 decay rate, average NDVI did not affect the likelihood that pests exceeded economic thresholds in any generation. Finally, effects of vineyards and NDVI on pest infestations, outbreaks and insecticide sprays strengthened when each variable was assessed in isolation (i.e. in models with no other landscape predictors; Table S3). However, when analysed separately, grasslands no longer predicted variation in pest outbreaks, insecticide applications or infestation rates (except in the first generation).

## DISCUSSION

As in other studies (Rusch *et al*., 2017; Papura *et al*., 2020), our results demonstrate that surrounding landscapes influence *Lobesia botrana* infestations on farms. Specifically, our models predicted that the likelihood of exceeding established economic thresholds at harvest (i.e. generation 3) would increase four‐fold, from 2.5% in landscapes with no surrounding vineyard cover to 10% in landscapes with 90% vineyard cover. (Note: vineyard cover may be slightly underestimated in our dataset due to land‐use classification errors for small farms.) In contrast, more productive landscapes and landscapes with more surrounding shrub cover were less likely to experience outbreaks and trigger insecticide sprays.

Pest outbreaks may be more frequent in simpler landscapes both because expansive monocultures allow pest populations to rapidly build and disperse (Root, 1973; O’Rourke and Petersen, 2017) and because simpler landscapes often contain fewer resources to support natural enemies (Landis *et al*., 2000). However, complex landscapes may also provide alternative host plants for pests (Tscharntke *et al*., 2016). Ultimately, the net effect of landscape complexity on pests likely depends on a balance among crop resource, natural enemy and alternative host effects. Without manipulative experiments or data on natural enemies or alternative hosts, we can only speculate as to why *L. botrana* outbreaks increased in landscapes with more vineyards. That said, increased food availability likely facilitated *L. botrana* population growth and dispersal in vineyard‐dominated landscapes, causing populations to gradually build and pass economic thresholds as grapes matured (i.e. generations 2 and 3). If declines in natural enemy populations caused pest outbreaks to increase with surrounding vineyard cover, then we would have expected outbreaks to also increase in other simplified landscapes. Yet olive and cereal‐dominated landscapes exhibited no such trends.

Explaining contrasting effects of surrounding grasslands and shrublands on *L. botrana* is more difficult. Both habitat types may contain woody plants that act as alternative host plants for *L. botrana* during the winter (Thiéry and Moreau, 2005). Moverover, while non‐crop habitats are known to benefit some of *L. botrana*’s natural enemies (e.g. birds and harvestmen), we are not aware of studies that differentiate effects of shrublands versus grasslands (Rusch *et al*., 2017; Papura *et al*., 2020). Still, grasslands exhibit low productivity in Mediterranean ecosystems (i.e. NDVI) (Alcaraz‐Segura et al., 2009), landscape productivity is often linked with biodiversity (Radeloff *et al*., 2019) and natural enemy diversity tends to increase biocontrol (Dainese et al., 2019). Thus, it is possible that, in grasslands, positive effects of alternative host plants on *L. botrana* eclipse negative effects of natural enemies. Indeed, positive effects of grasslands on *L. botrana* were strongest early in the growing season, potentially indicating that *L. botrana* may overwinter in alternative host plants in grasslands before dispersing into vineyards. In contrast, negative effects of shrublands and landscape productivity were stronger later on (i.e. during generations 2 and 3), which could reflect natural enemy populations gradually dispersing into vineyards and ultimately mitigating *L. botrana* outbreaks later in the season.

We also documented complex relationships between pest infestations and insecticide applications. Farmers were much more likely to apply insecticides if economic thresholds were overcome at least once. However, the amount of time spent above the threshold yielded a saturating relationship with the number of times that farmers applied insecticides. One potential explanation for this trend may be that most farmers follow IPM guidelines, spraying insecticides after each instance that the pest exceeds the economic threshold. Others, however, may oppose insecticides and never spray, allowing pests to build and remain above economic thresholds throughout the season. Indeed, after excluding farms that never sprayed any insecticides, we observed a more linear relationship between the fraction of time spent above the economic threshold and the number of insecticide sprays. Regardless of reason, our work highlights the importance of tracking multiple dimensions of pest control to guide decisions, as higher pest infestation rates do not necessarily translate into increased application rates (Chaplin‐Kramer *et al*., 2019). Further research into the factors driving farmers’ pest management decisions is all the more critical given recent European Union policy goals of halving pesticide use by 2030 (European Commission, 2020).

Despite variability in farmer behaviour, we still found that effects of landscape composition on insecticide applications mirrored landscape effects on pest outbreaks. Insecticide application frequencies were approximately twice as high on farms surrounded by 90% vineyard cover compared to farms with no vineyards in the surrounding landscape. On the surface, this trend could simply reflect variation in farmer values (e.g. conservation‐minded farmers may choose to conserve surrounding non‐crop habitats and refrain from applying insecticides). However, effects persisted after excluding farmers that never applied any insecticides, indicating that landscape effects on insecticide applications may be more directly tied to spatial variation in pest infestations.

These results suggest that landscape simplification could have cascading ramifications not only for farm yields but also for environmental and human health (Tilman *et al*., 2002; Foley *et al*., 2005). However, whether landscape simplification increases insecticide use in other cropping systems remains unclear. Several studies report that more insecticides are applied in areas with less crop diversity and surrounding semi‐natural habitat (Meehan *et al*., 2011; Larsen and Noack, 2017). But inconsistent relationships between landscape simplification and insecticides have been observed in corn and soybean‐producing USA states (Larsen, 2013, but see also Meehan and Gratton, 2015), suggesting landscape effects on insecticides may be crop or pest dependent.

Importantly, our study highlights the utility of ‘ecoinformatic’ analyses of long‐term, large‐scale datasets for landscape pest‐control studies (Rosenheim and Gratton, 2017). Pest infestation rates, outbreaks and insecticide applications were quite stochastic between years and across space, as reflected in the high level of deviance explained by random effects. Nonetheless, utilising a government‐sponsored database with large numbers of sites repeatedly surveyed across many years allowed us to identify sizable landscape effects that may have otherwise been masked by this variability. For less well‐replicated studies, teasing out landscape effects from stochastic noise is difficult and may explain why so many studies yield context‐dependent effects of surrounding landscape composition on pest populations (Karp *et al*., 2018). Indeed, reducing our database to sample sizes more reflective of traditional landscape studies substantially impeded our ability to detect landscape trends.

Overall, our results suggest that simplified landscapes increase vineyard pest outbreaks and escalate insecticide spray frequencies. In contrast, vineyards surrounded by more productive habitats and more shrubland area are less likely to exceed economic thresholds and apply insecticides. The implications of these results are relevant to diverse stakeholders. For farmers, our work demonstrates that increasing landscape complexity reduces the likelihood of suffering pest outbreaks, thus, mitigating costs associated with yield losses and insecticide applications. For government, NGOs and the public, our work highlights the importance of landscape planning for mitigating public and environmental impacts associated with insecticides (Pimentel *et al*., 1992).

At an individual level, farmers may be able to better control *L. botrana* populations through planting native vegetation in and around their farms. Ultimately, however, increasing landscape complexity will require coordinating groups of neighbouring farmers to maintain and/or restore productive, shrubland habitats. Although costly, formalised compensation programmes could help coordinate farmers to increase landscape complexity (Batáry *et al*., 2015). Such landscape‐informed coordination in pest management is an important advance needed to realise concrete co‐benefits for vineyard production, farmland conservation and human health.

## CONTRIBUTIONS

D.P., D.S.K., J.A.R., R.C.K. and S.W. designed research; D.P., D.S.K. and J.A.R analysed the data; D.P. and D.S.K. led the writing of the manuscript; All authors contributed critically to subsequent drafts.

### Peer Review

The peer review history for this article is available at https://publons.com/publon/10.1111/ele.13622.

### Open Research Badges

This article has earned Open Data badge. Data is available at (https://doi.org/10.25338/B84W6Z).

## Supporting information

Supplementary Material

## Data Availability

Data available via the Dryad Digital Repository (https://doi.org/10.25338/B84W6Z).
