# Supplementary material for: Landscape simplification increases vineyard pest outbreaks and insecticide use
Source: Ecol Lett. 2020 Oct 13;24(1):73–83. doi: 10.1111/ele.13622 (PMC7756857; doi:10.1111/ele.13622)

|  |  | AIC | |
| --- | --- | --- | --- |
| Generation | Decay Rate | Infestation | Economic threshold |
| 1 | 1250 | **709.29** | **337.22** |
| 1 | 750 | 711.99 | **337.20** |
| 1 | 250 | 714.97 | **337.22** |
| 2 | 1250 | **2659.12** | **1460.56** |
| 2 | 750 | 2660.59 | **1461.02** |
| 2 | 250 | 2660.83 | 1466.24 |
| 3 | 1250 | 2891.96 | 1493.60 |
| 3 | 750 | **2885.55** | **1489.11** |
| 3 | 250 | 2895.56 | **1487.74** |

Table S1. AIC values of models for each generation and response variable. Lowest values of AIC within a difference of two units are bolded.

Table S2. AIC values of insecticide application models with different decay rates. Lowest values of AIC within a difference of two units are bolded.

|  | AIC |  |
| --- | --- | --- |
| Decay Rate | Insecticide applications | |
| 1250 | **2018.90** | |
| 750 | 2024.92 | |
| 250 | 2021.70 | |

|  |  | Vineyards | | | | Annual crops | | | | Olives | | |
| --- | --- | --- | --- | --- | --- | --- | --- | --- | --- | --- | --- | --- |
|  |  | p-value | AIC | Dev. Expl. | p-value | | AIC | Dev. Expl. | p-value | | AIC | Dev. Expl. |
| Likelihood of exceeding  economic threshold | Generation 1 | 0.2469000 | 337.22 | 75.10% | 0.0655500 | | 331.59 | 74.50% | 0.7842170 | | 339.30 | 74.90% |
|  | Generation 2 | **0.0228300** | **1460.56** | **58.30%** | **0.0450300** | | **1468.53** | **56.40%** | 0.9576900 | | 1467.76 | 58.50% |
|  | Generation 3 | **0.0000851** | **1493.60** | **61.30%** | **0.0001850** | | **1490.64** | **60.90%** | 0.3372140 | | 1492.70 | 62.30% |
| Infestation rates | Generation 1 | **0.0219100** | **711.99** | **68.30%** | **0.0399700** | | **705.85** | **68.10%** | 0.4278150 | | 715.66 | 67.80% |
|  | Generation 2 | **0.0019900** | **2659.12** | **69.40%** | **0.0384300** | | **2666.82** | **68.90%** | 0.9743200 | | 2670.54 | 69.00% |
|  | Generation 3 | **0.0002510** | **2891.96** | **75.20%** | **0.0150310** | | **2896.20** | **74.80%** | 0.2844970 | | 2893.77 | 75.00% |
| Insecticide applications | All | **0.0001740** | **2033.50** | **52.80%** | **0.0083600** | | **2040.39** | **52.30%** | 0.3426000 | | 2044.79 | 53.20% |

Table S3. Comparisons of three sets of models, each including a different agricultural land-cover predictor (*i.e.,* surrounding vineyard, annual crop, and olive orchard cover). Oliver orchards never explained significant variation in pest outbreaks, infestations, or insecticide applications. Vineyards explained more variation in all three variables than annual crops (*i.e.,* more deviance explained and lower AIC). Significant predictors are bolded.

Table S4. Effects of landscape, topographic, and climate predictors, as well as random effects, on the likelihood of exceeding economic thresholds for each of the three generations of *Lobesia botrana*. The R^2^ and deviance explained by each model are also included. Significant values are bolded (P< 0.05).

|  | Generation 1 | Generation 2 | | Generation 3 |
| --- | --- | --- | --- | --- |
|  | p-value | p-value | p-value | |
| Average NDVI (Decay=1250) | 0.3402 | **0.0355** | 0.0919 | |
| % Forest (Decay=1250) | 0.9096 | 0.2747 | 0.7383 | |
| % Shrublands (Decay=1250) | 0.5487 | 0.3650 | 0.0721 | |
| % Grasslands (Decay=1250) | **0.0104** | 0.3006 | 0.2741 | |
| % Vineyards (Decay=1250) | 0.2469 | **0.0228** | **0.0001** | |
| Altitude | 0.1593 | 0.9596 | 0.0650 | |
| Aspect | 0.1298 | 0.0614 | 0.0945 | |
| Temperature PC1  (Autumn, winter, and spring temperatures) | 0.8523 | **0.0017** | 0.0664 | |
| Temperature PC2  (Summer temperatures) | **0.0205** | 0.7698 | 0.1250 | |
| Precipitation PC1  (Winter, spring and summer precipitations) | 0.2041 | **0.0212** | **0.0003** | |
| Precipitation PC2  (Autumn precipitations) | 0.4743 | 0.4566 | 0.1566 | |
| Technician (Random effect) | 0.1461 | **0.0049** | **0.0000** | |
| Cultivar (Random effect) | 0.7401 | **0.0125** | 0.4109 | |
| Year (Random effect) | **0.0001** | **0.0099** | **0.0000** | |
| Vineyard identity (Random effect) | **0.0028** | **0.0252** | **0.0160** | |
| Region (Random effect) | **0.0192** | 0.6622 | **0.0635** | |
| R^2^ | 0.665 | 0.494 | 0.549 | |
| Deviance Explained | 75.10% | 58.30% | 61.30% | |

Table S5. Effects of landscape, topographic, and climate predictors, as well as random effects, on pest infestation rates for each of the three generations of *Lobesia botrana*. The R^2^ and deviance explained by each model are also included. Significant values are bolded (P< 0.05).

|  | Generation 1 | Generation 2 | Generation 3 |
| --- | --- | --- | --- |
|  | p-value | p-value | p-value |
| Average NDVI (Decay=1250) | 0.7993 | 0.4287 | 0.1699 |
| % Forest (Decay=1250) | 0.2426 | 0.2602 | 0.6056 |
| % Shrublands (Decay=1250) | **0.0498** | 0.3377 | **0.0455** |
| % Grasslands (Decay=1250) | **0.0056** | **0.0377** | **0.0127** |
| % Vineyards (Decay=1250) | **0.0219** | **0.0020** | **0.0003** |
| Number of farm-years (weight) | **0.0006** | 0.3179 | **0.0645** |
| Altitude | 0.8864 | 0.7771 | **0.0326** |
| Aspect | 0.1137 | 0.3527 | 0.3567 |
| Temperature PC1  (Autumn, winter, and spring temperatures) | 0.6577 | 0.1825 | 0.3907 |
| Temperature PC2  (Summer temperatures) | **0.0299** | 0.6735 | 0.3796 |
| Precipitation PC1  (Winter, spring and summer precipitations) | **0.0125** | **0.0027** | **0.0002** |
| Precipitation PC2  (Autumn precipitations) | 0.4813 | 0.2267 | **0.0473** |
| Technician (Random effect) | **0.0000** | **0.0071** | **0.0004** |
| Cultivar (Random effect) | **0.0458** | **0.0058** | **0.0058** |
| Year (Random effect) | **0.0001** | **0.0019** | **0.0000** |
| Vineyard identity (Random effect) | **0.0381** | **0.0045** | **0.0002** |
| Region (Random effect) | 0.0584 | 0.5681 | **0.0234** |
| R^2^ | 0.694 | 0.641 | 0.666 |
| Deviance explained | 68.30% | 69.40% | 75.20% |

Table S6. Effect of exceeding economic thresholds at least once per year on the number of insecticide applications targeted to *Lobesia botrana*. The R^2^ and deviance explained by each model are also included. Significant values are bolded (P< 0.05).

|  | Generation 1 |
| --- | --- |
|  | p-value |
| Whether or not the threshold was exceeded at least once | **<0.0001** |
| Number of farm-years (weight) | 0.1922 |
| Technician (Random effect) | 0.0937 |
| Cultivar (Random effect) | 0.8580 |
| Year (Random effect) | 0.3417 |
| Vineyard identity (Random effect) | 0.8834 |
| Region (Random effect) | **<0.0001** |
| R^2^ | 0.545 |
| Deviance explained | 51.20% |

Table S7. Effect of the fraction of farm visits above the economic threshold on the number of insecticide applications targeted to *Lobesia botrana*. The R^2^ and deviance explained by each model are also included. Significant values are bolded (P< 0.05).

|  | Generation 1 |
| --- | --- |
|  | p-value |
| Proportion of farm visits exceeding the threshold | **<0.0001** |
| Number of farm-years (weight) | 0.0720 |
| Technician (Random effect) | **0**.**0147** |
| Cultivar (Random effect) | 0.4679 |
| Year (Random effect) | 0.4051 |
| Vineyard identity (Random effect) | 0.9756 |
| Region (Random effect) | **<0.0001** |
| R^2^ | 0.559 |
| Deviance explained | 52.20% |

Table S8. Effect of the fraction of farm visits above the economic threshold on the number of insecticide applications targeted to *Lobesia botrana*, after excluding farms where no pesticides were applied. The R^2^ and deviance explained by each model are also included. Significant values are bolded (P< 0.05).

|  | Generation 1 |
| --- | --- |
|  | p-value |
| Proportion of farm visits exceeding the threshold | **<0.0001** |
| Number of farm-years (weight) | 0.5109 |
| Technician (Random effect) | **0.0045** |
| Cultivar (Random effect) | 0.3825 |
| Year (Random effect) | 0.0507 |
| Vineyard identity (Random effect) | 0.9967 |
| Region (Random effect) | **0.0019** |
| R^2^ | 0.368 |
| Deviance explained | 40.40% |

Table S9. Effects of landscape, topographic, and climate predictors, as well as random effects, on the number of insecticide applications targeted to *Lobesia botrana*. The R^2^ and deviance explained by each model are also included. Significant values are bolded (P< 0.05).

|  | p-value |
| --- | --- |
| Average NDVI (Decay=1250) | 0.9760 |
| % Forest (Decay=1250) | 0.6051 |
| % Shrublands (Decay=1250) | **0.0222** |
| % Grasslands (Decay=1250) | 0.0533 |
| % Vineyards (Decay=1250) | **0.0002** |
| Number of farm-years (weight) | 0.2011 |
| Altitude | 0.1915 |
| Aspect | 0.5999 |
| Temperature PC1  (Autumn. winter. and spring temperatures) | 0.8444 |
| Temperature PC2  (Summer temperatures) | 0.2492 |
| Precipitation PC1  (Winter. spring and summer precipitations) | 0.2194 |
| Precipitation PC2  (Autumn precipitations) | 0.9238 |
| Technician (Random effect) | **0.0065** |
| Cultivar (Random effect) | 0.3848 |
| Year (Random effect) | **0.0166** |
| Vineyard identity (Random effect) | **0.0130** |
| Region (Random effect) | **<0.0001** |
| R^2^ | 0.549 |
| Deviance explained | 52.80% |

Table S10. Effects of landscape, topographic, and climate predictors, as well as random effects, on pest infestation rates (fraction of grape bunches infested) for the three generations of *Lobesia botrana*. Here, models with landscape variables using smaller scales are presented (decay rates of 250 and 750). Significant values are bolded (P< 0.05).

|  |  | Decay = 250 |  |  |  | Decay = 750 |  |
| --- | --- | --- | --- | --- | --- | --- | --- |
|  | Generation 1 | Generation 2 | Generation 3 |  | Generation 1 | Generation 2 | Generation 3 |
|  | p-value | p-value | p-value |  | p-value | p-value | p-value |
| Average NDVI | 0.7564 | 0.4618 | **0.0395** |  | 0.8998 | 0.4416 | 0.0913 |
| % Forest | 0.6761 | 0.6926 | 0.2898 |  | 0.3407 | 0.4429 | 0.2015 |
| % Shrublands | 0.8876 | 0.3972 | 0.1764 |  | 0.0908 | 0.3230 | 0.0575 |
| % Grasslands | **0.0351** | 0.6412 | 0.0857 |  | **0.0035** | 0.0891 | **0.0114** |
| % Vineyards | **0.0472** | **0.0031** | **0.0061** |  | **0.0172** | **0.0043** | **0.0009** |
| Number of farm-years (weight) | 0.0032 | 0.3251 | 0.0815 |  | **0.0009** | 0.3206 | 0.0658 |
| Altitude | 0.7150 | 0.9262 | 0.0547 |  | 0.9102 | 0.8606 | **0.0361** |
| Aspect | 0.1470 | 0.1405 | 0.1943 |  | 0.1070 | 0.2495 | 0.2899 |
| Temperature PC1  (Autumn, winter, and spring temperatures) | 0.9645 | 0.1463 | 0.4887 |  | 0.5886 | 0.1531 | 0.3681 |
| Temperature PC2  (Summer temperatures) | 0.0868 | 0.7925 | 0.2038 |  | **0.0489** | 0.7337 | 0.3370 |
| Precipitation PC1  (Winter, spring and summer precipitations) | **0.0135** | **0.0026** | **0.0003** |  | **0.0084** | **0.0027** | **0.0002** |
| Precipitation PC2  (Autumn precipitations) | 0.7773 | 0.2416 | **0.0410** |  | 0.6561 | 0.2363 | **0.0415** |
| Technician (Random effect) | **0.0000** | **0.0088** | **0.0011** |  | **0.0000** | **0.0068** | **0.0005** |
| Cultivar (Random effect) | **0.1000** | **0.0025** | **0.0013** |  | **0.0676** | **0.0059** | **0.0055** |
| Year (Random effect) | **0.0000** | **0.0017** | **0.0000** |  | **0.0001** | **0.0017** | **0.0000** |
| Vineyard identity (Random effect) | **0.0063** | **0.0017** | **0.0001** |  | **0.0251** | **0.0026** | **0.0001** |
| Region (Random effect) | 0.1219 | 0.6517 | **0.0301** |  | 0.0784 | 0.6056 | **0.0263** |

Table S11. Effects of landscape, topographic, and climate predictors, as well as random effects, on the likelihood of exceeding economic thresholds for the three generations of *Lobesia botrana*. Here, models with landscape variables using smaller scales are presented (decay rates of 250 and 750). Significant values are bolded (P< 0.05).

|  |  | Decay = 250 |  |  | |  | | Decay = 750 | |  | |
| --- | --- | --- | --- | --- | --- | --- | --- | --- | --- | --- | --- |
|  | Generation 1 | Generation 2 | Generation 3 |  | Generation 1 | | Generation 2 | | Generation 3 | |  |
|  | p-value | p-value | p-value |  | p-value | | p-value | | p-value | |  |
| Average NDVI | 0.2719 | 0.2515 | 0.3060 |  | 0.4740 | | **0.0451** | | 0.0985 | |  |
| % Forest | 0.6262 | 0.6866 | 0.5186 |  | 0.9806 | | 0.3247 | | 0.4975 | |  |
| % Shrublands | 0.3514 | 0.3492 | 0.1395 |  | 0.2055 | | 0.2302 | | 0.0521 | |  |
| % Grasslands | **0.0368** | 0.7482 | 0.1106 |  | **0.0165** | | 0.4368 | | 0.2819 | |  |
| % Vineyards | 0.0967 | **0.0244** | **0.0059** |  | 0.1779 | | **0.0332** | | **0.0017** | |  |
| Altitude | 0.1172 | 0.8108 | 0.1423 |  | 0.1417 | | 0.8880 | | 0.0886 | |  |
| Aspect | 0.1219 | **0.0464** | 0.0852 |  | 0.1375 | | **0.0483** | | 0.0841 | |  |
| Temperature PC1  (Autumn, winter, and spring temperatures) | 0.7878 | **0.0152** | 0.1070 |  | 0.8910 | | **0.0016** | | 0.0693 | |  |
| Temperature PC2  (Summer temperatures) | **0.0273** | 0.9143 | 0.1492 |  | **0.0208** | | 0.8228 | | 0.1493 | |  |
| Precipitation PC1  (Winter, spring and summer precipitations) | 0.1320 | **0.0234** | **0.0005** |  | 0.1567 | | **0.0236** | | **0.0003** | |  |
| Precipitation PC2  (Autumn precipitations) | 0.6020 | 0.3384 | 0.1062 |  | 0.5664 | | 0.4377 | | 0.1515 | |  |
| Technician (Random effect) | 0.1485 | **0.0286** | **0.0000** |  | 0.1613 | | **0.0058** | | **0.0000** | |  |
| Cultivar (Random effect) | 0.7161 | **0.0035** | **0.0471** |  | 0.7658 | | **0.0089** | | 0.2907 | |  |
| Year (Random effect) | **0.0000** | **0.0108** | **0.0000** |  | **0.0000** | | **0.0089** | | **0.0000** | |  |
| Vineyard identity (Random effect) | **0.0032** | **0.0290** | **0.0020** |  | **0.0013** | | **0.0273** | | **0.0050** | |  |
| Region (Random effect) | **0.0229** | 0.5487 | 0.0847 |  | **0.0239** | | 0.7137 | | 0.1065 | |  |

|  | Decay = 250 | Decay = 750 |
| --- | --- | --- |
|  | p-value | p-value |
| Average NDVI | 0.9132 | 0.2755 |
| % Forest | 0.4164 | 0.1328 |
| % Shrublands | **0.0492** | 0.9096 |
| % Grasslands | 0.1170 | 0.2013 |
| % Vineyards | **0.0007** | **0.0107** |
| Number of farm-years (weight) | 0.2086 | 0.2309 |
| Altitude | 0.2278 | 0.4970 |
| Aspect | 0.5957 | 0.4735 |
| Temperature PC1  (Autumn, winter, and spring temperatures) | 0.7253 | 0.4170 |
| Temperature PC2  (Summer temperatures) | 0.2438 | 0.1956 |
| Precipitation PC1  (Winter, spring and summer precipitations) | 0.2159 | 0.1920 |
| Precipitation PC2  (Autumn precipitations) | 0.8947 | 0.6421 |
| Technician (Random effect) | **0.0066** | **0.0141** |
| Cultivar (Random effect) | 0.3842 | 0.5581 |
| Year (Random effect) | **0.0157** | **0.0125** |
| Vineyard identity (Random effect) | **0.0036** | **0.0062** |
| Region (Random effect) | **<0.0001** | **0.0030** |

Table S12. Effects of landscape, topographic, and climate predictors, as well as random effects, on the number of insecticide applications targeted to *Lobesia botrana*. Here, models with landscape variables using smaller scales are presented (decay rates of 250 and 750). Significant values are bolded (P< 0.05).

|  |  | Decay = 250 |  |  |  | Decay = 750 |  |  |  | Decay = 1250 |  |
| --- | --- | --- | --- | --- | --- | --- | --- | --- | --- | --- | --- |
|  | Generation 1 | Generation 2 | Generation 3 |  | Generation 1 | Generation 2 | Generation 3 |  | Generation 1 | Generation 2 | Generation 3 |
|  | p-value | p-value | p-value |  | p-value | p-value | p-value |  | p-value | p-value | p-value |
| Average NDVI | 0.8523 | 0.4836 | 0.0480 |  | 0.8523 | 0.4836 | 0.0480 |  | 0.9532 | 0.5033 | 0.1920 |
| % Forest | 0.6712 | 0.8595 | 0.2482 |  | 0.6712 | 0.8595 | 0.2482 |  | 0.2726 | 0.2535 | 0.5515 |
| % Shrublands | 0.9241 | 0.3060 | 0.1673 |  | 0.9241 | 0.3060 | 0.1673 |  | 0.0654 | 0.1759 | 0.0381 |
| % Grasslands | 0.0802 | 0.5299 | 0.1656 |  | 0.0802 | 0.5299 | 0.1656 |  | 0.0208 | 0.0278 | 0.0350 |
| % Vineyards | 0.1009 | 0.0065 | 0.0264 |  | 0.1009 | 0.0065 | 0.0264 |  | 0.0340 | 0.0043 | 0.0011 |
| Number of farm-years (weight) | 0.0015 | 0.2953 | 0.0639 |  | 0.0015 | 0.2953 | 0.0639 |  | 0.0002 | 0.2800 | 0.0525 |
| Altitude | 0.9288 | 0.8461 | 0.0635 |  | 0.9288 | 0.8461 | 0.0635 |  | 0.7680 | 0.6777 | 0.0549 |
| Aspect | 0.0846 | 0.1255 | 0.2068 |  | 0.0846 | 0.1255 | 0.2068 |  | 0.0712 | 0.2831 | 0.3423 |
| # of insecticide applications | 0.0933 | 0.0001 | 0.0000 |  | 0.0933 | 0.0001 | 0.0000 |  | 0.1567 | 0.0001 | 0.0000 |
| # of herbicide applications | 0.5248 | 0.1430 | 0.1490 |  | 0.5248 | 0.1430 | 0.1490 |  | 0.7179 | 0.1095 | 0.1358 |
| # of tillage events | 0.7189 | 0.5949 | 0.8959 |  | 0.7189 | 0.5949 | 0.8959 |  | 0.8601 | 0.6159 | 0.9929 |
| # of fungicide applications | 0.2546 | 0.0349 | 0.2598 |  | 0.2546 | 0.0349 | 0.2598 |  | 0.1486 | 0.0375 | 0.2083 |
| Temperature PC1  (Autumn, winter and spring temperatures) | 0.9203 | 0.0118 | 0.3314 |  | 0.9203 | 0.0118 | 0.3314 |  | 0.6315 | 0.0166 | 0.2550 |
| Temperature PC2  (Summer temperatures) | 0.0209 | 0.8635 | 0.1688 |  | 0.0209 | 0.8635 | 0.1688 |  | 0.0104 | 0.9724 | 0.2744 |
| Precipitation PC1  (Winter, spring and summer precipitations) | 0.0234 | 0.0008 | 0.0009 |  | 0.0234 | 0.0008 | 0.0009 |  | 0.0245 | 0.0008 | 0.0007 |
| Precipitation PC2  (Autumn precipitations) | 0.7913 | 0.0389 | 0.0182 |  | 0.7913 | 0.0389 | 0.0182 |  | 0.5916 | 0.0471 | 0.0194 |
| Technician (Random effect) | 0.0001 | 0.0008 | 0.0003 |  | 0.0001 | 0.0008 | 0.0003 |  | 0.0000 | 0.0027 | 0.0001 |
| Cultivar (Random effect) | 0.1900 | 0.0005 | 0.0005 |  | 0.1900 | 0.0005 | 0.0005 |  | 0.1458 | 0.0017 | 0.0020 |
| Year (Random effect) | 0.0000 | 0.0017 | 0.0000 |  | 0.0000 | 0.0017 | 0.0000 |  | 0.0002 | 0.0016 | 0.0000 |
| Vineyard identity (Random effect) | 0.0344 | 0.0017 | 0.0000 |  | 0.0344 | 0.0017 | 0.0000 |  | 0.1249 | 0.0012 | 0.0000 |
| Region (Random effect) | 0.2816 | 0.6017 | 0.0561 |  | 0.2816 | 0.6017 | 0.0561 |  | 0.1508 | 0.5481 | 0.0299 |

Table S13. Effects of landscape, topographic, farm management, and climate predictors, as well as random effects, on pest infestation rates (fraction of grape bunches infested) for the three generations of *Lobesia botrana*. Here, models with farm management predictors are included (at all spatial scales; decay rates). Significant values are bolded (P< 0.05).

|  |  | Decay = 250 |  |  |  | Decay = 750 |  |  |  | Decay = 1250 |  |
| --- | --- | --- | --- | --- | --- | --- | --- | --- | --- | --- | --- |
|  | Generation 1 | Generation 2 | Generation 3 |  | Generation 1 | Generation 2 | Generation 3 |  | Generation 1 | Generation 2 | Generation 3 |
|  | p-value | p-value | p-value |  | p-value | p-value | p-value |  | p-value | p-value | p-value |
| Average NDVI | 0.4119 | 0.1920 | 0.3910 |  | 0.5854 | 0.2043 | 0.1887 |  | 0.4680 | 0.1803 | 0.2226 |
| % Forest | 0.8764 | 0.6793 | 0.4584 |  | 0.9827 | 0.4803 | 0.4307 |  | 0.8309 | 0.3716 | 0.7015 |
| % Shrublands | 0.4076 | 0.3149 | 0.1300 |  | 0.2369 | 0.1822 | 0.0437 |  | 0.5336 | 0.2639 | 0.0615 |
| % Grasslands | 0.4062 | 0.9710 | 0.1223 |  | 0.0657 | 0.4390 | 0.4244 |  | 0.0276 | 0.2906 | 0.3540 |
| % Vineyards | 0.3928 | 0.0995 | 0.0209 |  | 0.5680 | 0.1466 | 0.0095 |  | 0.6395 | 0.1122 | 0.0007 |
| Altitude | 0.1699 | 0.5935 | 0.2051 |  | 0.1767 | 0.5779 | 0.1583 |  | 0.1837 | 0.6250 | 0.1300 |
| Aspect | 0.0656 | 0.0277 | 0.0988 |  | 0.0686 | 0.0342 | 0.0871 |  | 0.0565 | 0.0428 | 0.1072 |
| # of insecticide applications | 0.5448 | 0.0000 | 0.0002 |  | 0.5475 | 0.0000 | 0.0004 |  | 0.5968 | 0.0000 | 0.0004 |
| # of herbicide applications | 0.6391 | 0.1462 | 0.6026 |  | 0.6739 | 0.1392 | 0.5969 |  | 0.6567 | 0.1283 | 0.5879 |
| # of tillage events | 0.3373 | 0.5220 | 0.2300 |  | 0.2974 | 0.5534 | 0.2389 |  | 0.4156 | 0.5609 | 0.2364 |
| # of fungicide applications | 0.0345 | 0.0012 | 0.5210 |  | 0.0247 | 0.0017 | 0.6351 |  | 0.0178 | 0.0018 | 0.6864 |
| Temperature PC1  (Autumn, winter. and spring temperatures) | 0.7696 | 0.0022 | 0.1146 |  | 0.8395 | 0.0016 | 0.0716 |  | 0.8213 | 0.0020 | 0.0915 |
| Temperature PC2  (Summer temperatures) | 0.0121 | 0.4919 | 0.3783 |  | 0.0103 | 0.6584 | 0.4476 |  | 0.0141 | 0.7377 | 0.4268 |
| Precipitation PC1  (Winter, spring and summer precipitations) | 0.1305 | 0.0009 | 0.0003 |  | 0.1422 | 0.0010 | 0.0003 |  | 0.1809 | 0.0009 | 0.0003 |
| Precipitation PC2  (Autumn precipitations) | 0.6759 | 0.0434 | 0.0720 |  | 0.5995 | 0.0555 | 0.1079 |  | 0.5284 | 0.0554 | 0.1078 |
| Technician (Random effect) | 0.0930 | 0.0000 | 0.0000 |  | 0.1568 | 0.0000 | 0.0000 |  | 0.1575 | 0.0000 | 0.0000 |
| Cultivar (Random effect) | 0.6616 | 0.0011 | 0.0143 |  | 0.6587 | 0.0028 | 0.1082 |  | 0.6627 | 0.0036 | 0.1238 |
| Year (Random effect) | 0.0001 | 0.0021 | 0.0000 |  | 0.0000 | 0.0010 | 0.0000 |  | 0.0001 | 0.0008 | 0.0000 |
| Vineyard identity (Random effect) | 0.0236 | 0.0048 | 0.0003 |  | 0.0156 | 0.0012 | 0.0016 |  | 0.0225 | 0.0014 | 0.0057 |
| Region (Random effect) | 0.0675 | 0.4894 | 0.1550 |  | 0.0620 | 0.5296 | 0.1205 |  | 0.0574 | 0.5070 | 0.0547 |

Table S14. Effects of landscape, topographic, farm management, and climate predictors, as well as random effects, on the likelihood of exceeding economic thresholds for the three generations of *Lobesia botrana*. Here, models with farm management predictors are included (at all spatial scales; decay rates). Significant values are bolded (P< 0.05).

Table S15. Effects of landscape, topographic, farm management, and climate predictors, as well as random effects, on the number of insecticide applications targeted to *Lobesia botrana*. Here, models with farm management predictors are included (at all spatial scales; decay rates). Significant values are bolded (P< 0.05).

|  | Decay = 250 | Decay = 750 | Decay = 1250 |
| --- | --- | --- | --- |
|  | p-value | p-value | p-value |
| Average NDVI | 0.7880 | 0.6710 | 0.1120 |
| % Forest | 0.4695 | 0.3208 | 0.1454 |
| % Shrublands | 0.0474 | 0.1125 | 0.8127 |
| % Grasslands | 0.0505 | 0.0955 | 0.1739 |
| % Vineyards | 0.0004 | 0.0017 | 0.0201 |
| Number of farm-years (weight) | 0.4348 | 0.4277 | 0.4299 |
| Altitude | 0.1230 | 0.1428 | 0.3953 |
| Aspect | 0.5876 | 0.5853 | 0.4846 |
| # of insecticide applications | 0.4896 | 0.4938 | 0.4683 |
| # of herbicide applications | 0.1870 | 0.1464 | 0.1120 |
| # of tillage events | 0.0000 | 0.0000 | 0.0000 |
| # of fungicide applications | 0.9303 | 0.8075 | 0.3528 |
| Temperature PC1  (Autumn, winter, and spring temperatures) | 0.2423 | 0.2408 | 0.1791 |
| Temperature PC2  (Summer temperatures) | 0.2183 | 0.2212 | 0.2053 |
| Precipitation PC1  (Winter, spring and summer precipitations) | 0.6477 | 0.6201 | 0.4343 |
| Precipitation PC2  (Autumn precipitations) | 0.0291 | 0.0314 | 0.0286 |
| Technician (Random effect) | 0.4143 | 0.4315 | 0.5141 |
| Cultivar (Random effect) | 0.0014 | 0.0015 | 0.0017 |
| Year (Random effect) | 0.0155 | 0.0088 | 0.0149 |
| Vineyard identity (Random effect) | 0.0004 | 0.0005 | 0.0016 |

**Figure S1. *Lobesia botrana* generations over a growing season**. *L. botrana* completes three generations over a growing season, with infestation rates (% of grape inflorescences or bunches infested with eggs) increasing from one generation to the next. (Panel A) Line represents predictions from a Generalized Additive Mixed Model (GAMM); shaded region corresponds to 95% confidence region. (Panel B) Raw infestation data.


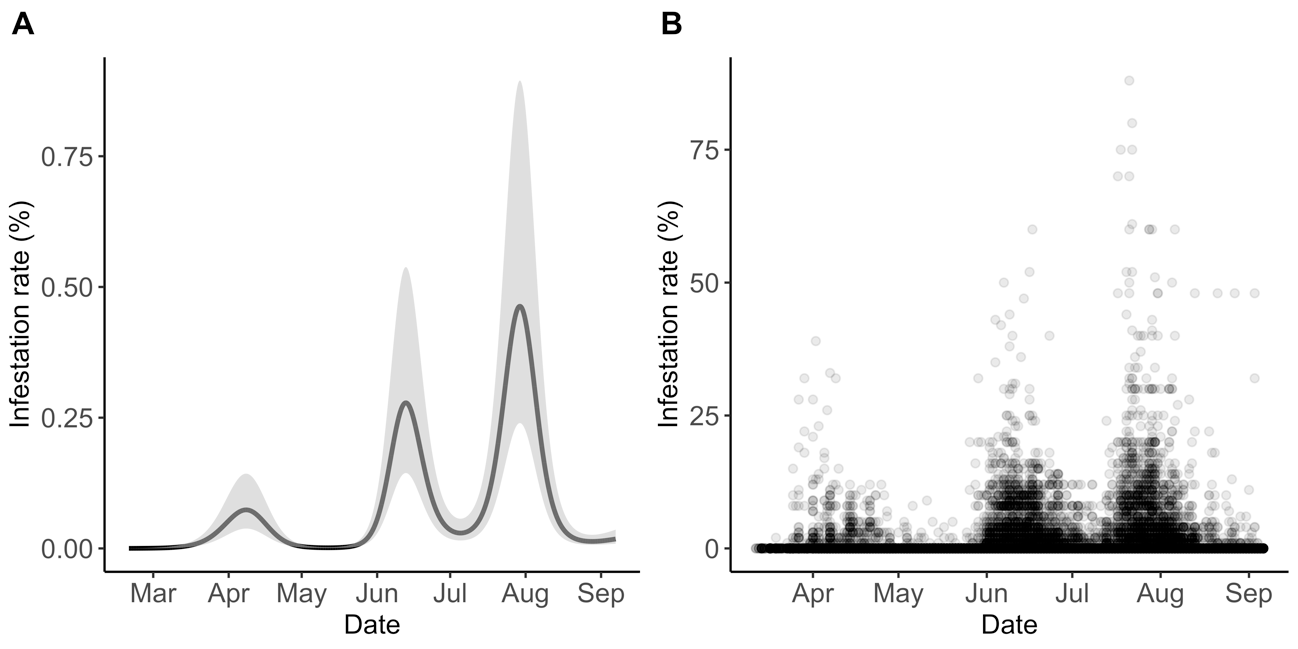


Figure S2. Scatterplot showing the relationship between the percentage of surrounding vineyards and the percentage of surrounding annual crops.


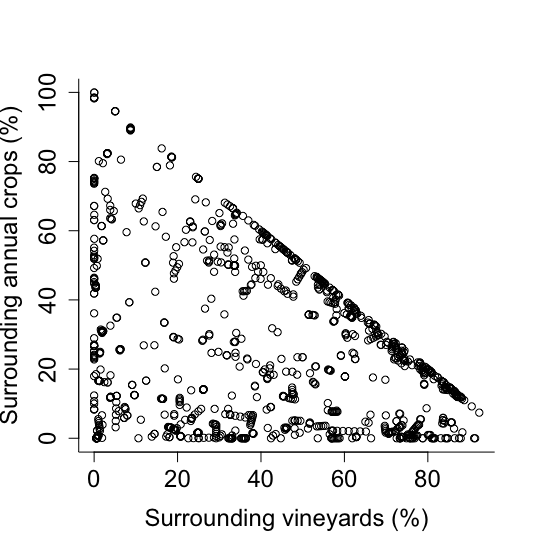


**Figure S3.** **Landscape effects on pest infestations (% of grape bunches with egg masses) for each generation of *Lobesia botrana***. Pest infestation rates increased with surrounding grassland cover in every generation (top panels; yellow lines) and decreased with surrounding shrubland cover in the first and third generations (middle panels; green lines). Infestations increased with surrounding vineyard cover in every generation (bottom panels; purple lines). Lines represent predictions from GAMMs; shaded regions correspond to 95% confidence regions.


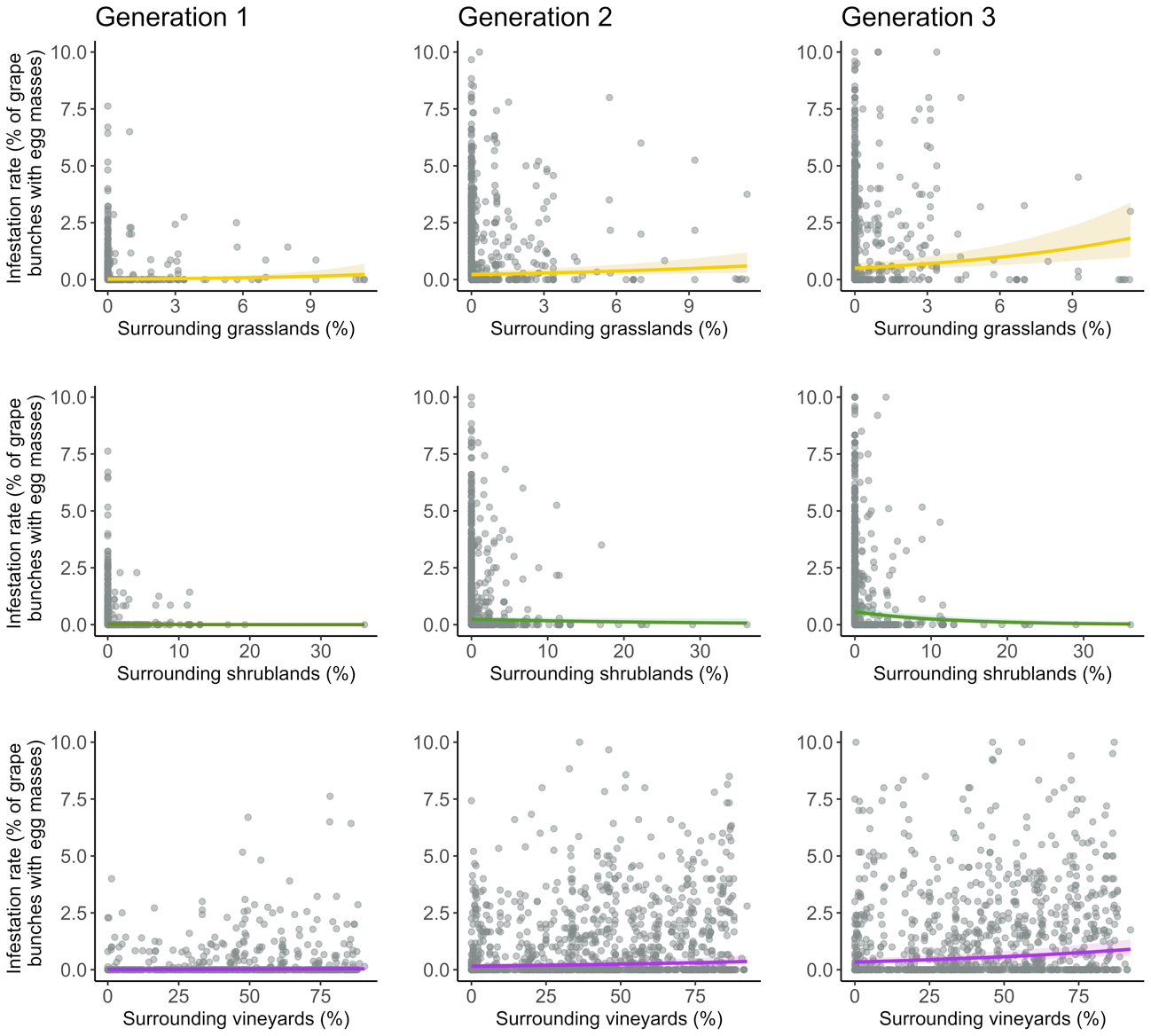


**Figure S4. Climate effects on the likelihood of exceeding economic thresholds.** Only significant effects are displayed (P<0.05). (Panel A) In second generation, the likelihood of exceeding economic thresholds decreased with hotter autumn, winter, and spring temperatures (Temperature Principal Component Axis 1). (Panel B) In first generation, the likelihood of exceeding economic thresholds decreased with hotter summer temperatures (Temperature PC Axis 2). In the second (Panel C) and third (Panel D) generations. economic thresholds were most likely to be exceeded at low to intermediate levels of winter. spring and summer precipitation (Precipitation PC Axis 1). Lines represent predictions from GAMMs; shaded regions correspond to 95% confidence regions.


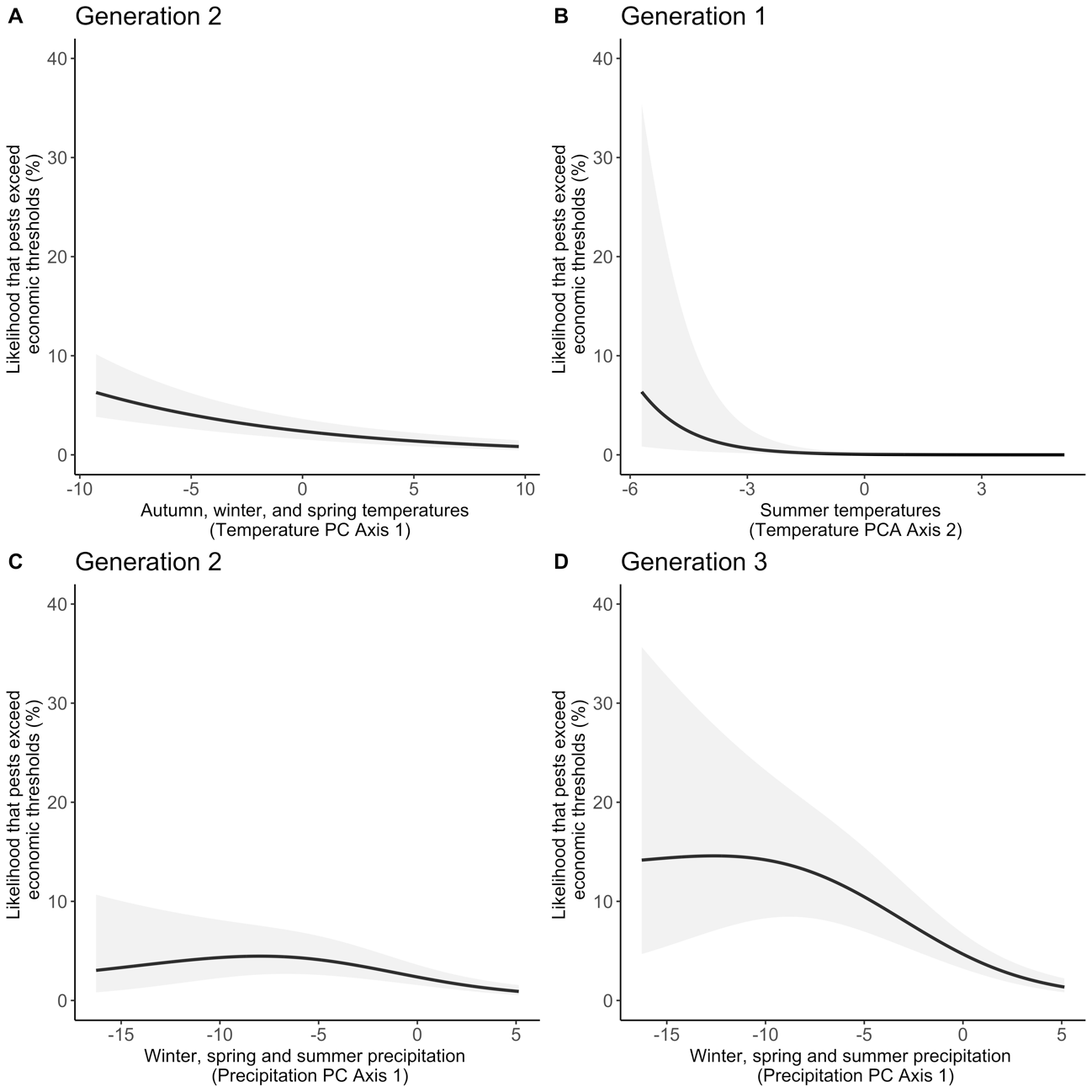


**Figure S5.** **Landscape effects on pest infestations (% of grape bunches with egg masses) for each generation of *Lobesia botrana* in models including management variables**. Lines represent predictions from GAMMs; shaded regions correspond Pest infestation rates increased with surrounding grassland cover in every generation (top panels) and decreased with surrounding shrubland cover in the third generation (middle panels). Infestations increased with surrounding vineyard cover in every generation (bottom panels). Lines represent predictions from GAMMs; shaded regions correspond to 95% confidence regions.


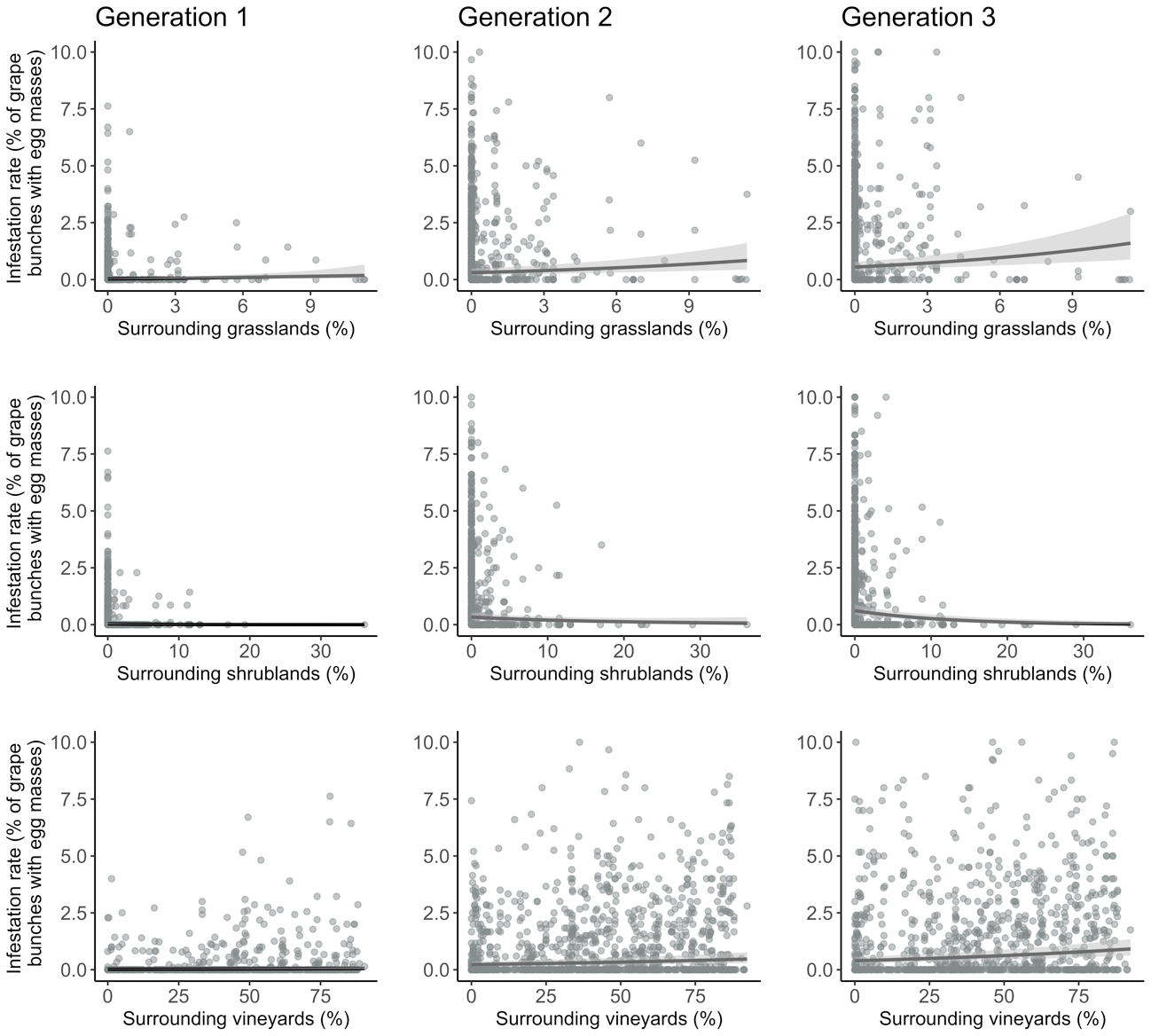


**Figure S6. Landscape effects on the likelihood of pests exceeding economic thresholds for each generation of *Lobesia botrana* in models including management variables**. Thresholds were more likely to be exceeded in landscapes with more surrounding grasslands in the first but not the second or third generations (top panels). In contrast, farms in landscapes with more vineyard cover were predicted to experience more outbreaks in the third generation. but not the first or second (middle panels). Finally, no effects of the Normalized Difference Vegetation Index were detected (bottom panels). Lines represent predictions from GAMMs; shaded regions correspond to 95% confidence regions.


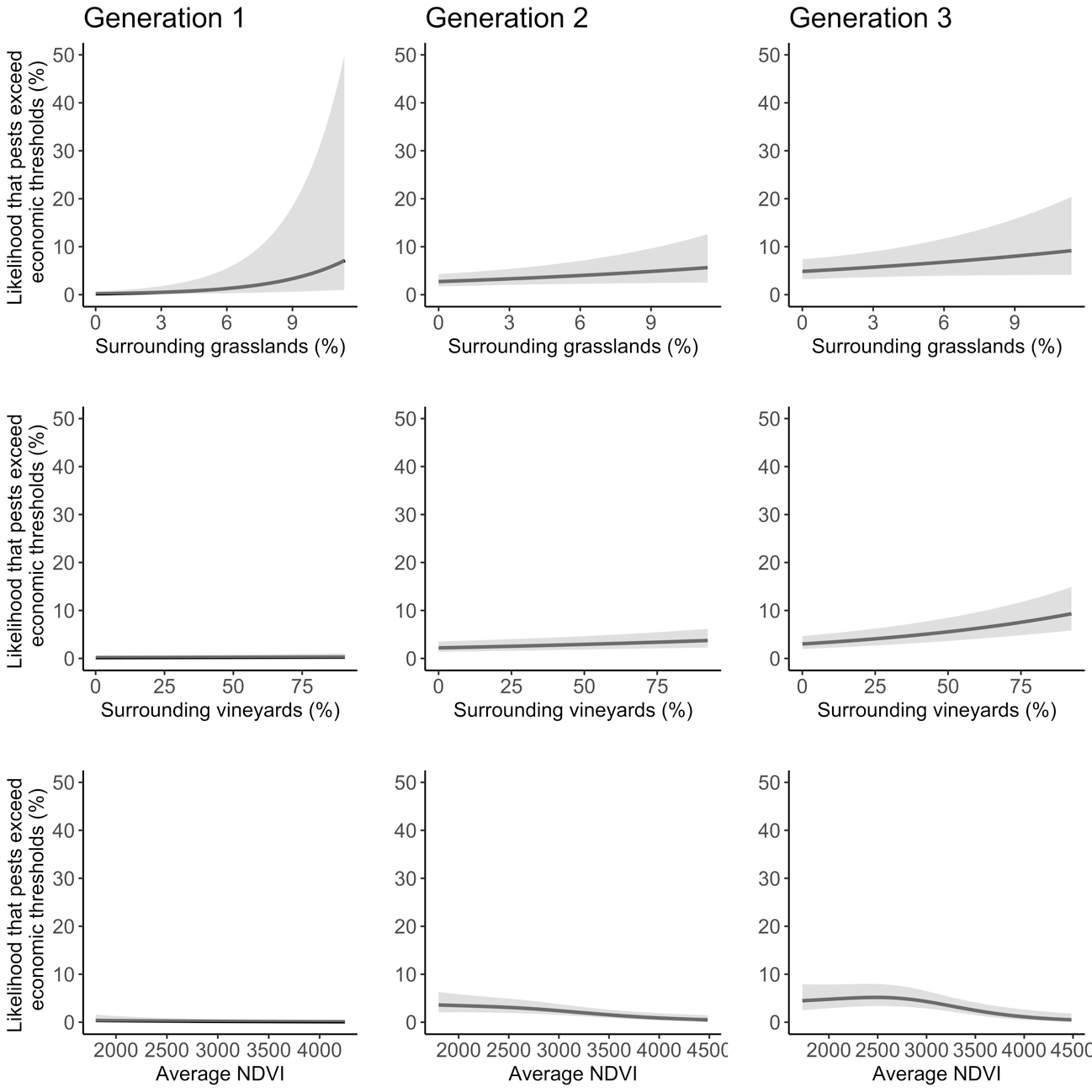


**Figure S7. Landscape effects on insecticide application rates, including management variables**. Farmers were more likely to spray insecticides in landscapes with less surrounding shrubland (left panel). In contrast, farmers applied more insecticides in landscapes with more surrounding vineyards (right panel). Lines represent predictions from GAMMs; shaded regions correspond to 95% confidence regions.


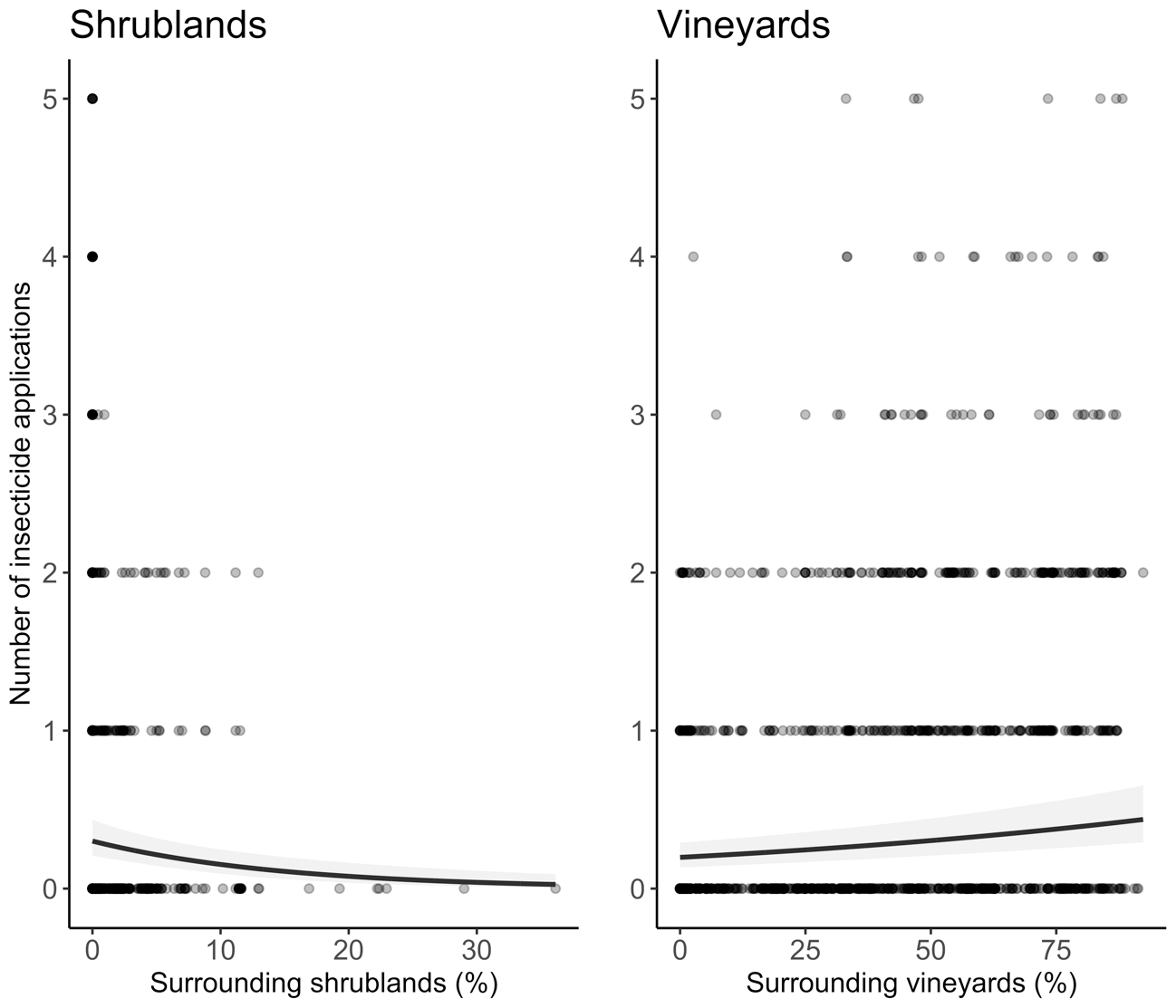

Supplement: Supplementary file 1 — Supplementary Material [file ELE-24-73-s001.docx]
